# MIRIS – an adequate method to measure fortified breast milk?

**DOI:** 10.1186/2194-7791-1-S1-A9

**Published:** 2014-09-11

**Authors:** Alexandra Kreissl, Valentina Zwiauer, Andreas Repa, Christoph Binder, Margarita Thanhaeuser, Angelika Berger, Nadja Haiden

**Affiliations:** Department of Pediatrics, Division of Neonatology, Pediatric Intensive Care Medicine and Neuropediatrics, Medical University of Vienna, Vienna, Austria

## Introduction

Human milk (HM) is unique and the gold standard for feeding neonates. HM contains insufficient quantity of protein for preterm infants. Therefore, a new supplement (Aptamil Protein+®-Milupa/Danone) was developed.

## Aim

The aim was to learn about the capability of MIRIS Human Milk Analyzer to measure the composition, particular of the protein content, in HM after addition of Human Milk Fortifier (HMF) and a bovine protein powder (P+®).

## Methods

Nutrient composition of HM was measured baseline, after fortification with commercial HMF (Aptamil 4.3%FMS®-Milupa/Danone and BEBA 5%FM85®-Nestlé), and after addition of further protein (P+®) in 0.5g steps up to 4.0g/100ml. Different milk processing types and the influence on MIRIS measurements was evaluated.

## Results

Preanalytic testing: MIRIS was capable to measure bovine protein dissolved in water accurately. Impreciseness started after adding 0.5g P+® to HM-fortifier mixtures and revealed up to 20% difference after addition of 4.0g P+®. Native HM (FMS group, n=67) contained in mean 1.1±0.3g protein, 3.1±0.8g fat, 6.6±0.3g lactose, 11.7±0.8g of dry mass and 59±10kcal per 100ml HM. After fortification of HM with HMF and 4.0g P+® a deviation in protein content of 1.0g protein/100ml in FMS® group and in FM85® group was observed. MIRIS underestimated protein content of HM fortified with HMF and P+® mixtures (p<0.05). MIRIS underevaluated carbohydrates and energy content. Processing of HM had no effects on protein level. Lactationday correlated significantly with protein content (p<0.0001; r=-,4243).

## Conclusions

MIRIS is an adjuvant tool to provide the actual nutrient composition of unfortified HM. MIRIS has the ability to measure solely bovine protein (in water) and human protein in HM. A combination of both types of protein generated imprecise results of protein content altogether. Processing had no effect on protein content. Protein significantly decreased with lactation period in preterm HM.

